# Supplementary material for: Trust Formation, Error Impact, and Repair in Human–AI Financial Advisory: A Dynamic Behavioral Analysis
Source: Behav Sci (Basel). 2025 Oct 7;15(10):1370. doi: 10.3390/bs15101370 (PMC12561693; doi:10.3390/bs15101370)
Supplement: Supplementary file 1 [file behavsci-15-01370-s001.zip › behavsci-3864150-supplementary.pdf]

Supplementary Material; S1

Supplement S1. Stimuli and on-screen flow. This reports the full scripts used for each condition (advisory type, product risk, single-error message, and post-error explanation), including exact wording and formatting.

Study 1.

- Group 1: Low-risk product with human expert recommendation
- Group 2: Low-risk product with AI robo-advisor recommendation
- Group 3: High-risk product with human expert recommendation
- Group 4: High-risk product with AI robo-advisor recommendation

1) Situation

Group 1 & 2; Left / Group 3 & 4; Right

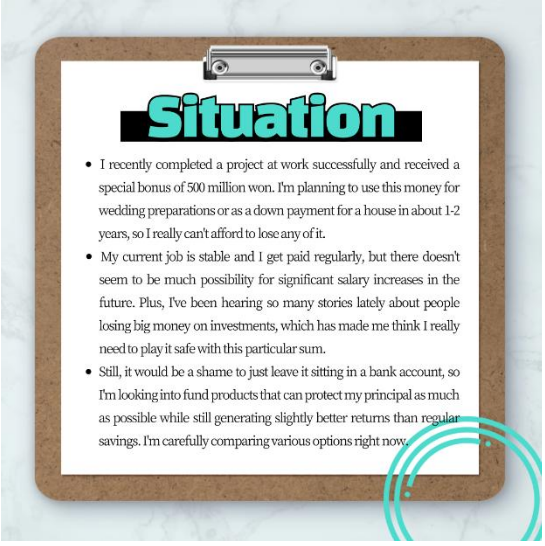

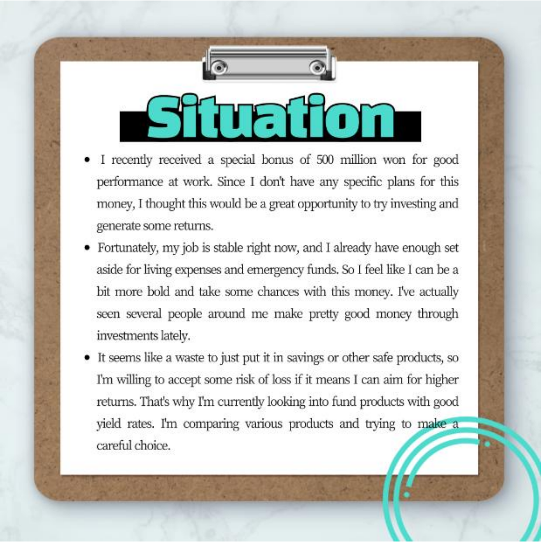

2) Product

Group 1 & 2

	① Best One	② Stellar Growth	③ Phoenix Rising	④ Quantum Leap	⑤ Aurora Investment	⑥ Pinnacle Equity	⑦ Triumph Capital
Fund type	Bond fund	Bond fund	Bond fund	Bond fund	Mixed bond fund	Bond fund	Bond fund
Net asset value (NAV)	1037.81 KRW	1064.43 KRW	1050.04 KRW	1059.27 KRW	1097.12 KRW	1007.32 KRW	1038.91 KRW
Total fund size (All classes combined)	2.94B KRW (197.4B KRW)	31M KRW (0.8B KRW)	213M KRW (4.3B KRW)	35.7B KRW (1,622B KRW)	10M KRW (19M KRW)	8M KRW (-)	1.26B KRW (12.23B KRW)
Initial sales fee	0.150%	0.100%	0.300%	0.150%	0.500%	0.000%	0.100%
Exit fee	None	None	None	None	None	None	None
Total expenses ratio	0.215%	0.42%	0.305	0.2195%	0.19	0.429%	0.2%
1-year return	7.78%	4.35%	5.21%	7.88%	3.75%	3.89%	4.28%
3-year return	16.99%	11.10%	11.64%	16.10%	6.27%	9.44%	10.49%
Fund rating (1-year)	★★★★☆	★★☆☆☆	★★★☆☆	★★★☆☆	★★★★☆	★★☆☆☆	★★☆☆☆
Risk level	Low risk	Low risk	Low risk	Low risk	Low risk	Low risk	Low risk

Group 3 & 4

	① Atlantic Bridge	② Golden Gate	③ Renaissance Portfolio	④ Power up	⑤ Liberty Capital	⑥ Meridian Global	⑦ Constellation
Fund type	Stock fund	Stock fund	Stock fund	Stock fund	Stock fund	Stock fund	Stock fund
Net asset value (NAV)	1432.74 KRW	1065.39 KRW	1368.59 KRW	1483.8 KRW	1630.99 KRW	1184.56 KRW	1004.03 KRW
Total fund size (All classes combined)	279M KRW (9.2B KRW)	1.5B KRW (118.5B KRW)	3.3B KRW (176.7B KRW)	30.6B KRW (240.3B KRW)	13.5B KRW (120.7B KRW)	1.3B KRW (25.4B KRW)	173M KRW (7.0B KRW)
Initial sales fee	0.500%	0.500%	0.500%	0.350%	0.450%	0.500%	0.500%
Exit fee	70%% of 1-90 day profits	None	None	None	None	None	None
Total expense ratio	1.11%	1.085%	1.154	0.918%	1.16%	1.19	0.775%
6-month return	19.37%	22.83%	19.08%	20.28%	36.07%	11.89%	17.24%
1-year return	15.22%	9.08%	10.12%	18.70%	21.87%	7.62%	15.60%
Fund rating (1-year)	★★★★★	★★★★☆	★★★★★	★★★★★	★★★★★	★★★★☆	★★★★★
Risk level	High risk	High risk	High risk	High risk	High risk	High risk	High risk

3) Recommendation

Group 1; Left / Group 2; Right

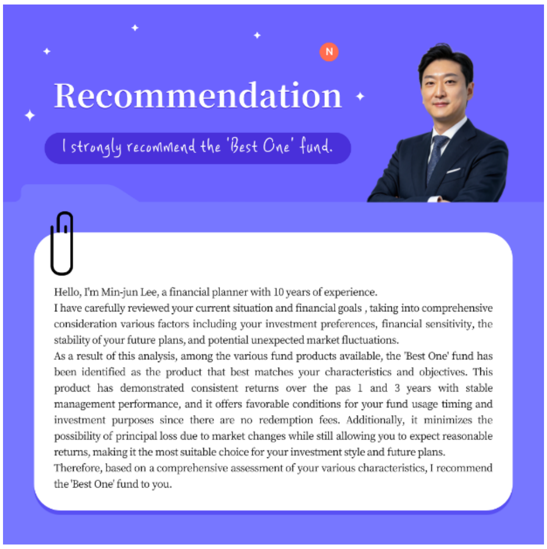

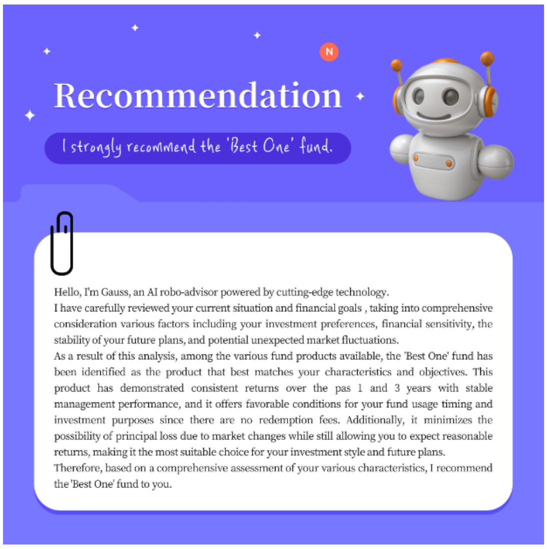

Group 3; Left / Group 4; Right

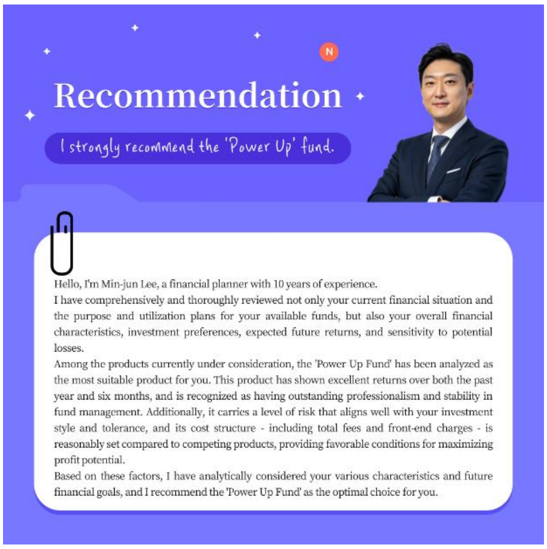

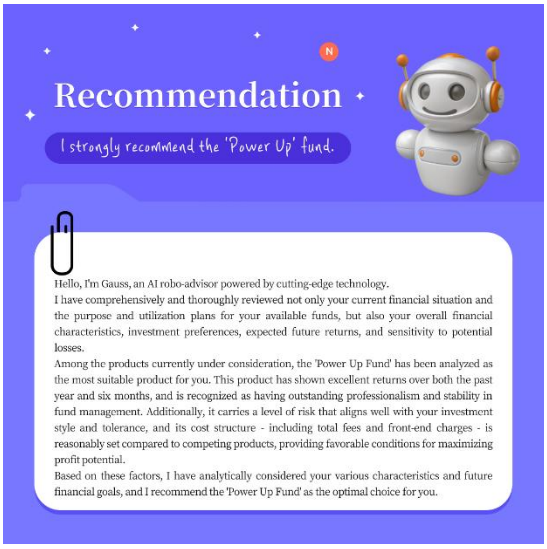

Study 2.

- Group 1: Human expert, accurate recommendations in all three rounds
- Group 2: Human expert, with an inaccurate recommendation in the second round and an explanation provided
- Group 3: Human expert, the inaccurate recommendation in the second round without explanation
- Group 4: AI robo-advisor, accurate recommendations in all three rounds
- Group 5: AI robo-advisor, with inaccurate recommendations in the second round and explanations provided
- Group 6: AI robo-advisor, the inaccurate recommendation in the second round without explanation

Round 1 Situation

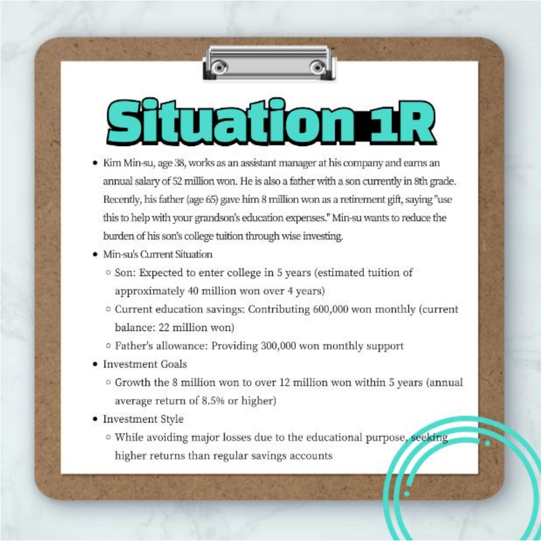

Round 1 Product

	① Diamond Select	② Sterling Balance	③ Royal Crown	④ Platinum Choice	⑤ Crystal Clear	⑥ Sapphire Elite	⑦ Harmony
Fund type	Stock fund	Stock fund	Mixed bond fund	Stock fund	Stock fund	Stock fund	Stock fund
Net asset value (NAV)	1388.78 KRW	1350.54 KRW	1201.91 KRW	1251.11 KRW	1397.28 KRW	1353.19 KRW	1445.05 KRW
Total fund size(All classes combined)	33.1B KRW (175.9B KRW)	3.6B KRW (14.6B KRW)	33.6B KRW (43.7B KRW)	929M KRW (8B KRW)	5.3B KRW (113.7B KRW)	21.8B KRW (26.5B KRW)	56M KRW (1.1B KRW)
Initial sales fee	0.500%	0.000%	0.000%	0.500%	0.500%	0.000%	0.500%
Exit fee	None	None	None	None	None	None	None
Total expense ratio	1.154%	1.72%	0.567	1.31%	0.938%	0.948	1.04%
6-month return	19.83%	20.00%	14.52%	13.00%	17.60%	17.76%	16.67%
1-year return	10.71%	11.80%	10.97%	9.16%	10.22%	10.15%	7.80%
Fund rating (1-year)	★★★★★	★★★★★	★★★★★	★★★★☆	★★★★★	★★★★★	★★★★☆
Asset composition (Domestic stocks)	97.38%	90.39%	33.64%	-	83.80%	84.74%	99.11%
Risk analysis (1-year) S.E. % ranking	22	62	90	83	34	41	67

Round 1 Recommendation

Group 1,2,3; Left / Group 4,5,6; Right

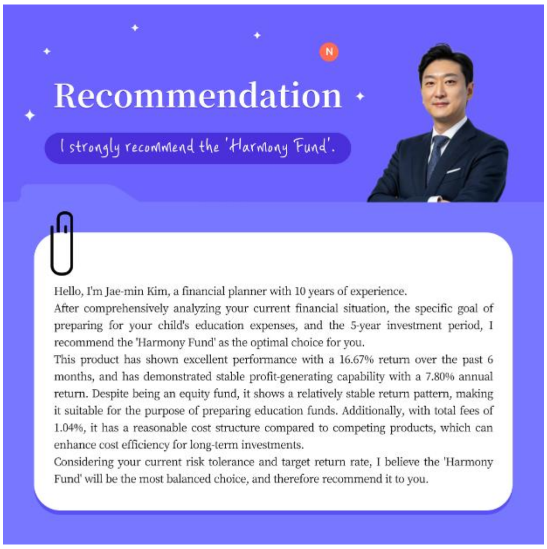

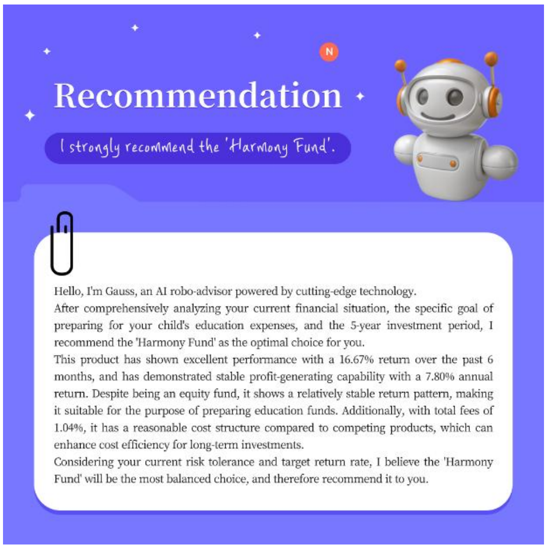

Round 1 Results

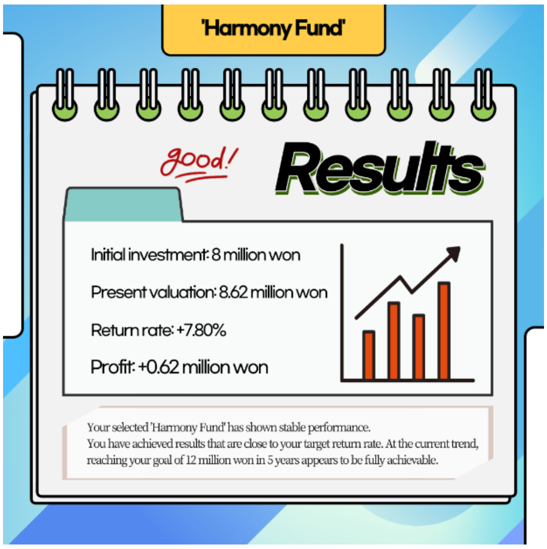

Round 2 Situation

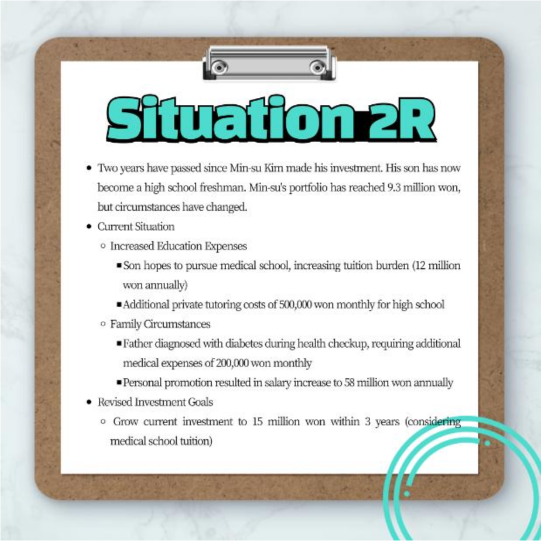

Round 2 Product

	① Thunder Storm	② Blue Wave	③ Lightning Strike	④ Hurricane Force	⑤ Tornado Spin	⑥ Tsunami Power	⑦ Cyclone Energy
Fund type	Stock fund	Stock fund	Stock fund	Stock fund	Stock fund	Fund of funds	Stock fund
Net asset value (NAV)	1464 KRW	2138.99 KRW	1029.9 KRW	12.5643 KRW	1448.68 KRW	2342.33 KRW	648.72 KRW
Total fund size(All classes combined)	331M KRW (9.9B KRW)	735M KRW (7.1B KRW)	173M KRW (7B KRW)	253M KRW (2.3B KRW)	31.7B KRW (384B KRW)	43M KRW (54M KRW)	12.4B KRW (41.3B KRW)
Initial sales fee	0.500%	1.000%	0.500%	0.000%	0.000%	0.500%	0.000%
Exit fee	70%% of 1-90 day profits	None	None	None	None	None	None
Total expense ratio	1.11%	1.46%	0.775	1.24%	1.43%	1.26	1.325%
6-month return	19.45%	29.38%	16.88%	7.88%	22.63%	19.35%	4.18%
1-year return	19.00%	18.28%	18.13%	16.64%	15.67%	15.31%	14.71%
Fund rating (1-year)	★★★★★	★★★★★	★★★★★	★★★★☆	★★★★★	★★★★★	★★★★☆
Asset composition (Domestic stocks)	76.26%	92.18%	95.07%	10.09%	92.93%	-	22.37%
Risk analysis (1-year) S.E. % ranking	3	94	4	85	5	35	77

Round 2 Recommendation

Group 1,2,3; Left / Group 4,5,6; Right

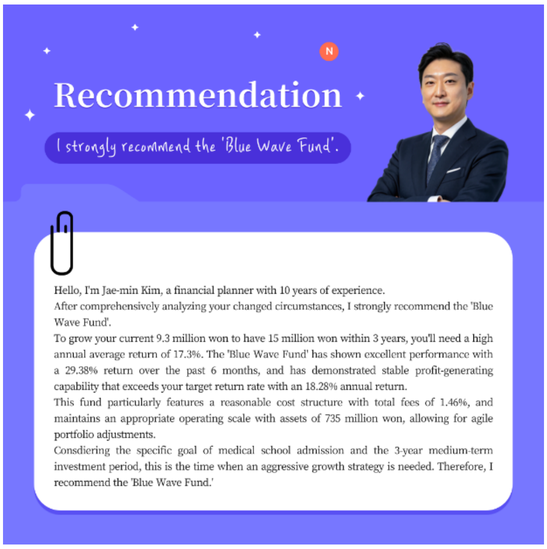

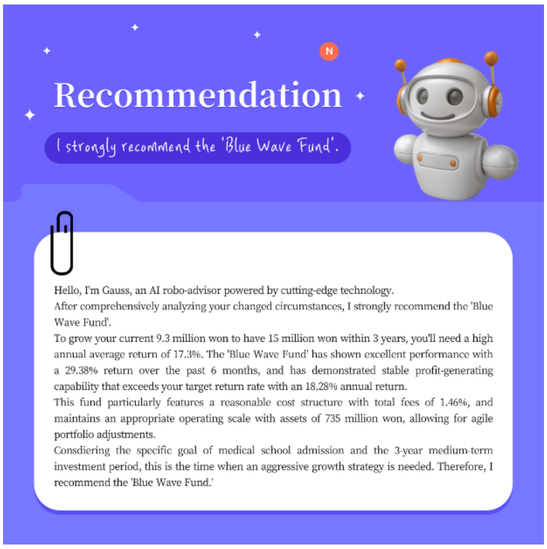

Round 2 Results

Group 1,4; Left / Group 2,3,5,6; Right

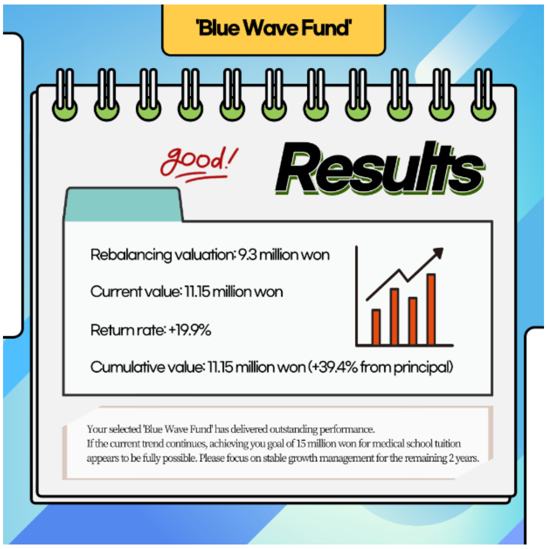

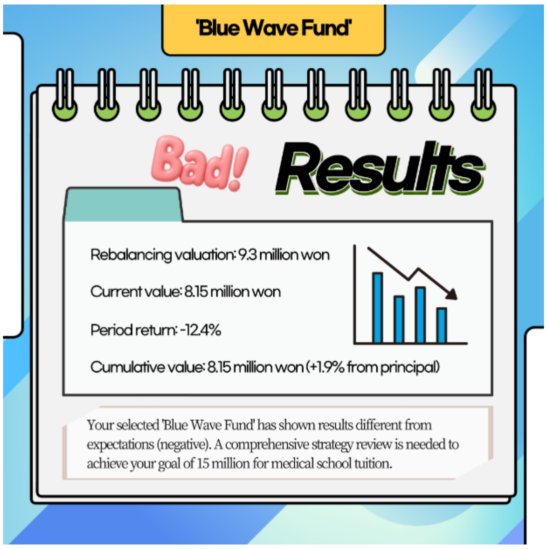

Round 2 Explanation

Group 2,5

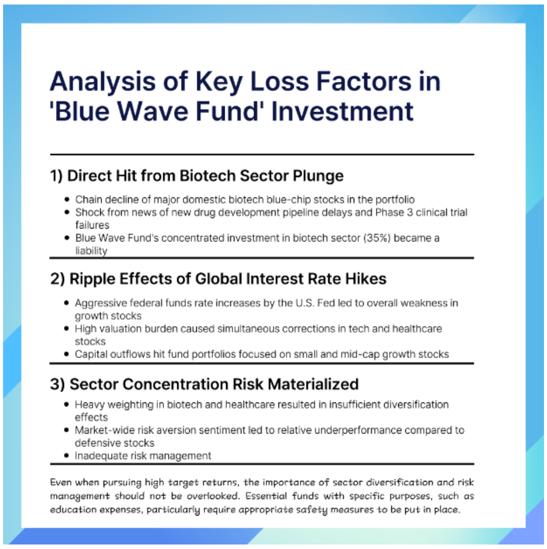

Round 3 Situation

Group 1,4; Left / Group 2,3,5,6; Right

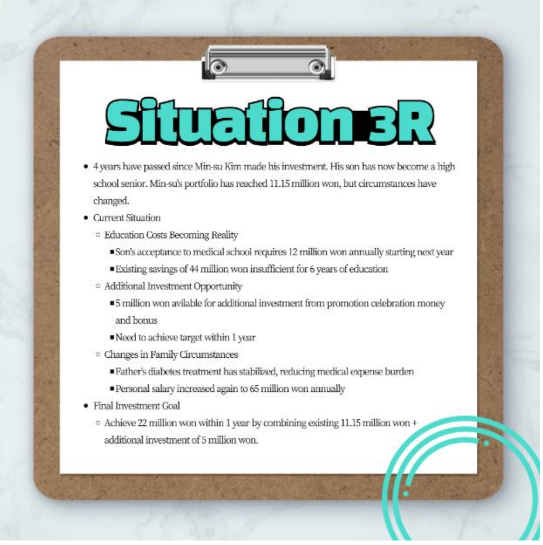

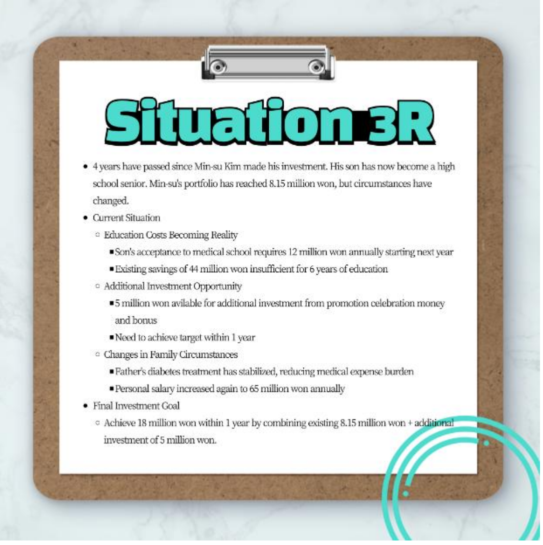

Round 3 Product

	① Rocket Launch	② Turbo Boost	③ Speed Demon	④ Flash Forward	⑤ Bullet Train	⑥ Jet Stream	⑦ Momentum
Fund type	Stock fund	Fund of funds	Fund of funds	Stock fund	Stock fund	Stock fund	Stock fund
Net asset value (NAV)	1397.28 KRW	1174.45 KRW	2342.33 KRW	1276.63 KRW	1214.84 KRW	1384.23 KRW	1431.6 KRW
Total fund size(All classes combined)	5.3B KRW (113.7B KRW)	1M KRW (297M KRW)	43M KRW (54M KRW)	19.7B KRW (881.4B KRW)	372M KRW (7.3B KRW)	143M KRW (1.5B KRW)	2.2B KRW (2.3B KRW)
Initial sales fee	0.500%	0.500%	0.500%	0.500%	0.000%	0.450%	0.500%
Exit fee	None	None	None	None	None	None	None
Total expense ratio	0.938%	1.41%	1.26	0.81%	0.8495%	0.29	0.672%
6-month return	17.60%	3.24%	19.35%	8.37%	13.83%	11.00%	7.29%
1-year return	10.22%	15.70%	15.31%	2.13%	4.56%	7.60%	16.87%
Fund rating (1-year)	★★★★★	-	★★★★★	★★★☆☆	★★★★☆	★★★★☆	-
Asset composition (Domestic stocks)	83.80%	-	-	99.39%	99.68%	85.62%	93.55%
Risk analysis (1-year) S.E. % ranking	34	73	35	9	75	1	-

Round 3 Recommendation

Group 1; Left / Group 4; Right

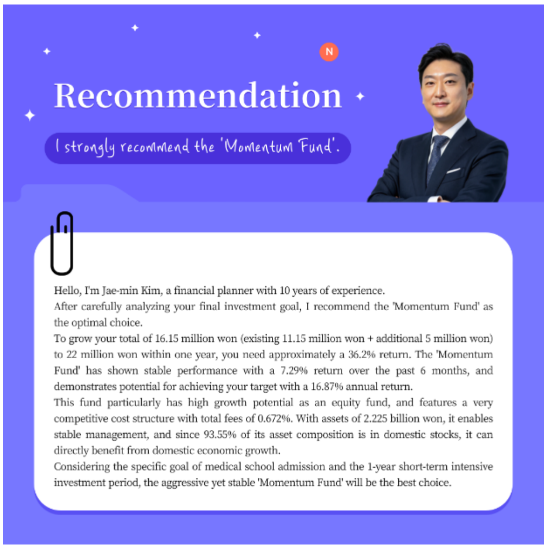

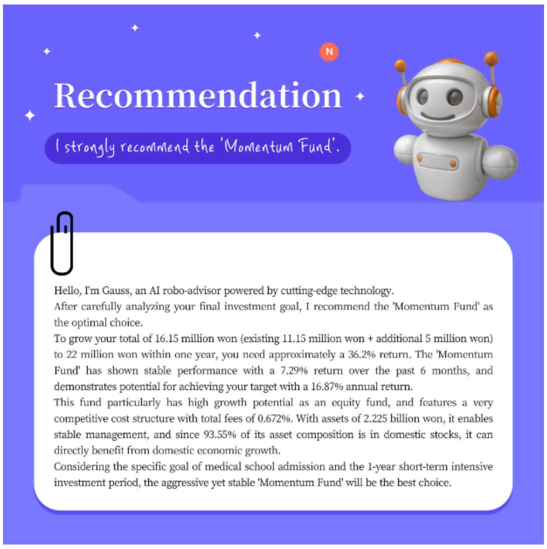

Group 2,3; Left / Group 5,6; Right

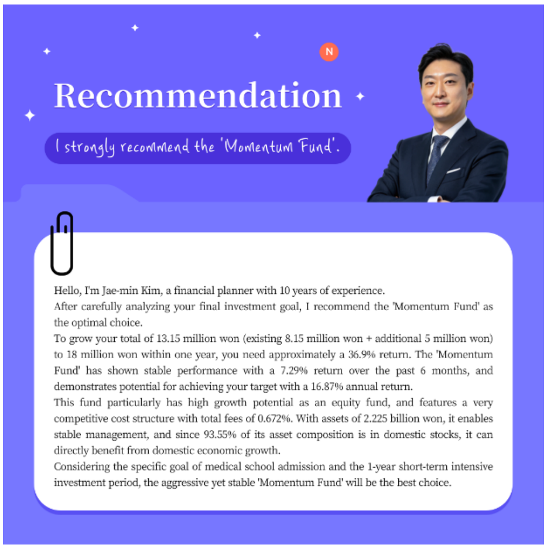

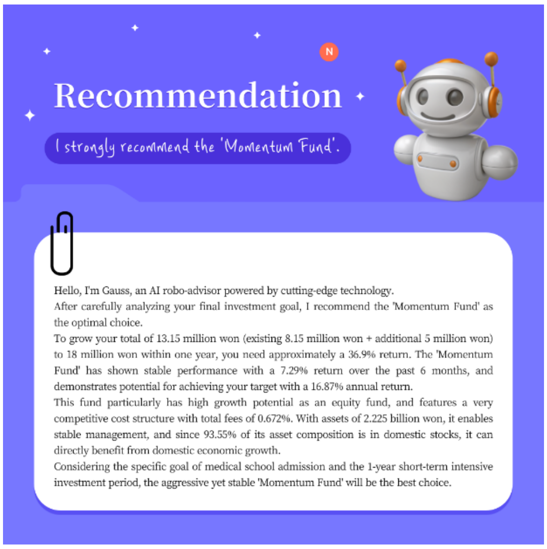

Round 3 Results

Group 1,4; Left / Group 2,3,5,6; Right

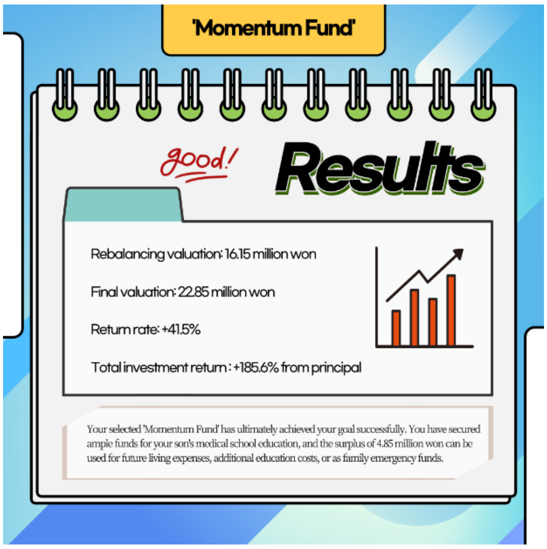

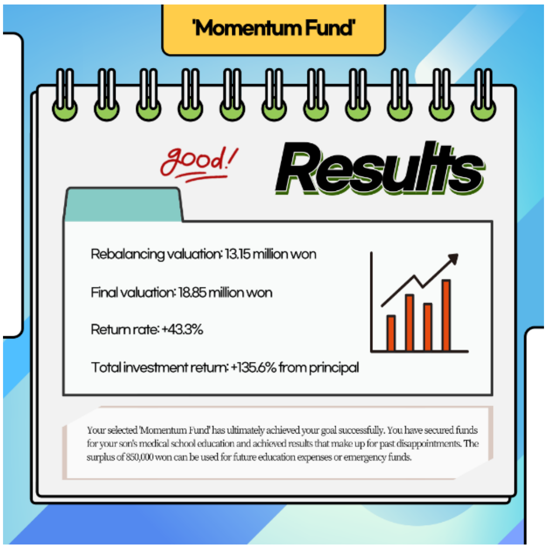

Supplementary Material; S2

Supplement S2. Measures, items, and reliability (Studies 1-2). Panel A summarizes item wordings, anchors, and reliability for Study 1; Panel B does so for Study 2. 'Reliance intention' in the main text corresponds to the instrument often labeled 'continued usage intention'; we retain the original items but report the construct as reliance in the text.

Panel A. Study 1

Construct	#Items	Sample item	Scale anchors	α (Study 1)	Source/notes
Risk attitude	1		0=not at all willing to take risks, …, 10=very willing to take risks	-	Dohmen et al. (2011)
Financial literacy	6	Interest compounding, …	finding the right answers	-	FINRA
Financial skill	9	I know how to make complex financial decisions.	1=Strongly disagree, …, 7=strongly agree	0.879	CFPB
Self-efficacy	5	I can always manage to solve difficult problems if I try hard enough.	Same	0.879	Schwarzer & Jerusalem(1995)
AI literacy	5	I can identify the AI technology employed in the applications and products I use.	Same	0.852	Wang et al. (2023)
Trust in AI	3	If I use the AI service, I think I would be able to depend on it completely.	Same	0.834	Gulati et al. (2019)
Trust	5	The recommendation information is believable.	Same	0.910	Appelman & Sundar (2015)
Satisfaction	1	I am overall satisfied with my decision-making based on the above recommendation information.	Same	-	-
Reliance intention*	3	I will continue to use the above recommendation information in the future.	Same	0.879	-

* 'Reliance intention' in the text corresponds to the instrument's 'continued usage intention' scale.

Panel B. Study 2

Construct	#Items	Sample item	Scale anchors	α (Study 1)	Source/notes
Risk attitude	1		0=not at all willing to take risks, …, 10=very willing to take risks	-	Dohmen et al. (2011)
Financial literacy	6	Interest compounding, …	finding the right answers	-	FINRA
Financial skill	9	I know how to make complex financial decisions.	1=Strongly disagree, …, 7=strongly agree	0.900	CFPB
Self-efficacy	5	I can always manage to solve difficult problems if I try hard enough.	Same	0.903	Schwarzer & Jerusalem(1995)
AI literacy	5	I can identify the AI technology employed in the applications and products I use.	Same	0.876	Wang et al. (2023)
Trust in AI	3	If I use the AI service, I think I would be able to depend on it completely.	Same	0.853	Gulati et al. (2019)
Trust	5	The recommendation information is believable.	Same	1R 0.923<br>2R 0.922<br>3R 0.927	Appelman & Sundar (2015)
Satisfaction	1	I am overall satisfied with my decision-making based on the above recommendation information.	Same	-	-
Reliance intention*	3	I will continue to use the above recommendation information in the future.	Same	1R 0.912<br>2R 0.906<br>3R 0.905	-

* 'Reliance intention' in the text corresponds to the instrument's 'continued usage intention' scale.

Measurements with Financial Literacy

1. Interest compounding: Suppose you had $100 in a savings account and the interest rate is 20% per year and you never withdraw money or interest payments. After 5 years, how much would you have on this account in total?
   - More than $200
   - Exactly $200
   - Less than $200
   - Do not know
2. Inflation: Imagine that the interest rate on your savings account was 1% per year and inflation was 2% per year. After 1 year, how much would you be able to buy with the money in this account?
   - More than today
   - Exactly the same
   - Less than today
   - Do not know
3. Time value of money: Assume a friend inherits $10,000 today and his sibling inherits $10,000 3 years from now. Who is richer because of the inheritance?
   - My friend
   - His sibling
   - They are equally rich
   - Do not know
4. Money illusion: Suppose that in the year 2026, your income has doubled and the prices of all goods have doubled too. In 2026, how much will you be able to buy with your income?
   - More than today
   - The same
   - Less than today

- Do not know

5. Risk diversification: Buying a single company's stock usually provides a safer return than a stock mutual fund.
   - True
   - False
   - Do not know
6. Bond pricing: If interest rates rise, what will typically happen to bond prices?
   - They will rise
   - They will fall
   - They will stay the same
   - There is no relationship between bond prices and the interest rates
   - Do not know

Measurements with variables

**Financial Skill**
- I know how to make complex financial decisions.
- I am able to make good financial decisions that are new to me.
- I know how to get myself to follow through on my financial intentions.
- I am able to recognize a good financial investment.
- I know how to keep myself from spending too much.
- I know how to make myself save.
- I know where to find the advice I need to make decisions involving money.
- I know when I need advice about my money.
- I have no trouble understanding financial information.
**Self-efficacy**
- I can always manage to solve difficult problems if I try hard enough.
- It is easy for me to stick to my aims and accomplish my goals.
- When I am confronted with a problem, I can usually find several solutions.
- I am confident that I could deal efficiently with unexpected events.
- Thanks to my resourcefulness, I know how to handle unforeseen situations.
**AI Literacy**

- I can identify the AI technology employed in the applications and products I use.
- I can skillfully use AI applications or products to help me with my daily work.
- I can choose the most appropriate AI application or product from a variety for a particular task.
- I always comply with ethical principles when using AI applications or products.
- I am always alert to the abuse of AI technology.
**Trust in AI**
- If I use the AI service, I think I would be able to depend on it completely.
- I can always rely on the AI service for decision-making.
- I can trust the information presented to me by the AI service.
**Trust in Recommendations**
- The recommendation information is believable.
- The recommendation information is factual.
- The recommendation information is credible.
- The recommendation information is trustworthy.
- The recommendation information is comprehensive.
**Satisfaction**
- I am overall satisfied with my decision-making based on the above recommendation information.
**Continued Usage Intention**
- I will continue to use the above recommendation information in the future.
- I will recommend the above recommendation information to others as well.
- I plan to use the above recommendation information in the long term.

Supplementary Material; S3

Supplement S3. Manipulation checks. Panel A summarizes checks for Study 1 (advisor identity, perceived risk). Panel B summarizes checks for Study 2 (perceived inaccuracy at Round 2; receipt of the post-error explanation). Where applicable, we report group differences (chi-squared tests or t-tests).

Panel A. Study 1

Check		Metric	Perceived		X^2
			Low-risk	High-risk	
Perceived Risk	Low	% correct	87 (93.5)	6 (6.5)	127.506***
	High		11 (11.5)	85 (88.5)	
			Human expert	AI robo-advisor	
Advisor Identity	Human	% correct	71 (78.0)	20 (22.0)	104.359***
	AI		5 (5.1)	93 (94.9)	

Notes: M = mean; SD = standard deviation; N = number of observations. *** $p < 0.001$

Panel B. Study 2

Check		Metric	Perceived		X^2
			Human expert	AI robo-advisor	
1R Advisor Identity	Human	% correct	123 (83.7)	24 (16.3)	147.327***
	AI		19 (12.9)	128 (87.1)	
			Human expert	AI robo-advisor	
2R Advisor Identity	Human	% correct	122 (83.0)	25 (17.0)	165.924***
	AI		12 (8.2)	135 (91.8)	
			Accurate	Inaccurate	
2R Perceived Accuracy		M ± SD	5.29 (1.05)	2.28 (1.43)	20.556***
			Provided	Not Provided	
2R Explanati on	Provided	% correct	72 (74.2)	25 (25.8)	27.070***
	Not Provided		36 (37.1)	61 (62.9)	
			Human expert	AI robo-advisor	
3R Advisor	Human	% correct	117 (79.6)	30 (20.4)	152.283***

Identity	AI	12 (8.2)	135 (91.8)

Notes: M = mean; SD = standard deviation; N = number of observations. *** $p < 0.001$

Supplementary Material; S4

Supplement S4. Robustness checks.

ANCOVA Results for Trust, Satisfaction, and Continued Usage Intention: Main and Interaction Effects with Covariates

Variable	Trust		Satisfaction		Reliance	
	F	Partial η^2	F	Partial η^2	F	Partial η^2
Intercept	12.595***	0.068	8.191**	0.045	2.778	0.016
Gender (male=0)	3.146	0.018	0.850	0.005	0.988	0.006
Age	0.088	0.001	1.760	0.010	2.510	0.014
Monthly income (KRW 10k)	4.299*	0.024	2.208	0.013	3.072	0.018
High school (university=0)	2.359	0.014	3.633	0.021	2.278	0.013
Graduate (university=0)	0.140	0.001	0.543	0.003	0.183	0.001
Risk Attitude	0.024	0.000	0.395	0.002	0.021	0.000
Financial Skill	3.308	0.019	1.891	0.011	3.396	0.019
Financial Literacy	0.655	0.004	3.487	0.020	4.633*	0.026
Self-efficacy	0.801	0.005	0.008	0.000	1.112	0.006
Investment Independence	0.062	0.000	0.292	0.002	0.253	0.001
Prospectus Review (don't=0)	8.079**	0.045	0.262	0.002	0.228	0.001
AI Literacy	1.778	0.010	4.650*	0.026	1.250	0.007
Trust in AI	14.346***	0.077	5.332*	0.030	13.930***	0.075
Risk Level	1.230	0.007	0.105	0.001	0.305	0.002
Advisory Type	6.714*	0.038	5.338*	0.030	10.485**	0.057
Interaction Terms	0.100	0.001	0.002	0.000	2.319	0.013
Adjusted R^2	0.235		0.168		0.275	

Note: Interaction terms represent Risk Level*Advisory Type; Significance level: * $p < 0.05$, ** $p < 0.01$, *** $p < 0.001$.

Supplementary Material; S5

Supplement S5. Robustness checks.

Linear Mixed-Effects Model Robustness Check Results

Hypothesis	Interaction Term	β	SE	t	p	95% CI
Trust						
H2: Single-error shock	2R × accuracy	.947***	.114	8.300	<.001	[0.724, 1.171]
	3R × accuracy	.068	.114	.590	.554	[-0.156, 0.291]
H3: Post-error repair	2R × explanation	.678***	.145	4.690	<.001	[0.395, 0.962]
	3R × explanation	.083	.145	.570	.568	[-0.201, 0.366]
H4: Literacy moderation	2R × financial literacy	-.094*	.045	-2.090	.037	[-0.182, -0.006]
	3R × financial literacy	.017	.045	0.380	.700	[-0.071, 0.105]
Satisfaction						
H2: Single-error shock	2R × accuracy	1.135***	.147	7.720	<.001	[0.847, 1.423]
	3R × accuracy	.236	.147	1.600	.109	[-0.053, 0.524]
Reliance						
H2: Single-error shock	2R × accuracy	1.061***	.131	8.120	<.001	[0.805, 1.317]
	3R × accuracy	.200	.131	1.530	.126	[-0.056, 0.456]

Notes: Linear mixed-effects models included participant-level random intercepts and all covariates from the original ANOVA models. Results confirm the robustness of key interaction effects. *p < .05, **p < .01, ***p < .001.
